# Rare Earth Elements Uptake by Synthetic Polymeric and Cellulose-Based Materials: A Review

**DOI:** 10.3390/polym14214786

**Published:** 2022-11-07

**Authors:** Gabriel Salfate, Julio Sánchez

**Affiliations:** Departamento de Ciencias del Ambiente, Facultad de Química y Biología, Universidad de Santiago de Chile, Santiago 9170022, Chile

**Keywords:** rare earth elements, lanthanides, cellulose, functional polymers, adsorption, ion exchange

## Abstract

Contemporary industrial processes and the application of new technologies have increased the demand for rare earth elements (REEs). REEs are critical components for many applications related to semiconductors, luminescent molecules, catalysts, batteries, and so forth. REEs refer to a group of 17 elements that have similar chemical properties. REE mining has increased considerably in the last decade and is starting an REE supply crisis. Recently, the viability of secondary REE sources, such as mining wastewaters and acid mine drainage (AMD), has been considered. A strategy to recover REEs from secondary water-related sources is through the usage of adsorbents and ion exchange materials in preconcentration steps due to their presence in low concentrations. In the search for more sustainable processes, the evaluation of synthetic polymers and natural source materials, such as cellulose-based materials, for REE capture from secondary sources should be considered. In this review, the chemistry, sources, extraction, uses, and environmental impact of REEs are briefly described to finally focus on the study of different adsorption/ion exchange materials and their performance in capturing REEs from water sources, moving from commercially available ion exchange resins to cellulose-based materials.

## 1. Introduction

**Figure 1:**
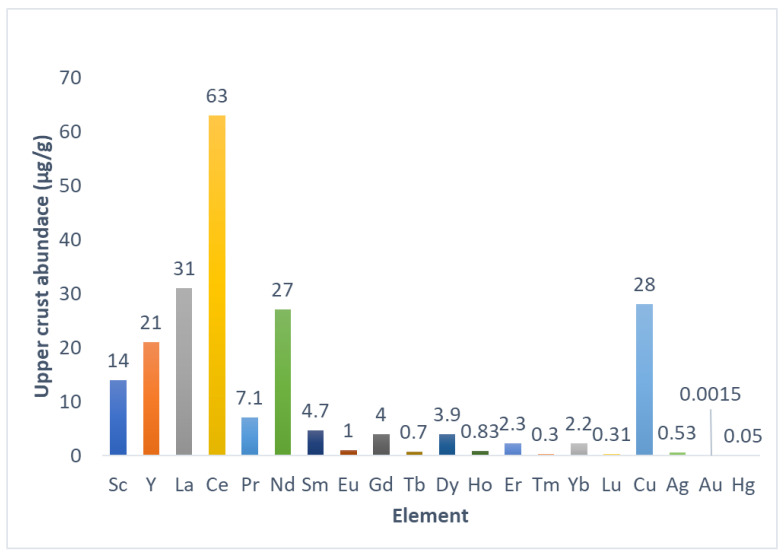
Abundance of REEs and semiprecious and precious metals in the earth’s upper crust. The graph was tabulated using information from [1].

Rare earth elements (REEs) refer to a group of 17 metals that include inner transition metals, also known as *f*-block elements or lanthanides, from the periodic table of elements together with scandium and yttrium [1,2,3]. It has been suggested that scandium should be excluded from REEs due to its chemical properties, which differ enough from other REEs [2,3,4]. Additionally, promethium is not a naturally occurring element, which excludes it from REEs [2,3,4,5]. Although their denomination suggests scarcity, REEs altogether are more abundant than Cu, Ag, Hg, and most precious metals [1,2,3]. Individually, the most abundant REE is Ce. La and Ce are more abundant than Cu, and Nd is as abundant as Cu [1,2,3]. The less abundant REEs individually are more abundant than Hg and Au [1,2,3] (Figure 1). However, abundance is not related to availability for exploitation due to REEs’ chemical properties, which differ from other metals [2,3,4,5]. Economically relevant deposits of REEs are found in China, Vietnam, Brazil, Russia, India, United States, Australia, Myanmar, and India [3,4]. REE-containing minerals and ores are processed through thermal and chemical treatments, and REEs are purified individually by means of fraction crystallization, solvent extraction, and/or ion exchange processes [2,3]. REEs have many applications due to their chemistry, which allows for obtaining materials with unique properties for highly specific applications. Petroleum, chemicals, electronics, high-technology devices, medicine, and agriculture use REE-based compounds and materials as critical components [3,4,5,6], making REEs technologically relevant. Additionally, the possibility of substitution of REEs for other elements is mostly limited to exchanging less abundant REEs for more abundant REEs in materials, and substituting them with non-REE elements is challenging due to the lesser efficiency of non-REE materials under development for the same applications [5]. Due to their importance, finding new sources of REEs is also critical. Despite their abundance, REEs’ distribution in the earth’s crust is dispersed, and rarely accumulates to form mineral ores and deposits, mainly because REE cations could substitute other ions in the crystal lattices of minerals [3,4]. Currently, REEs are mined using extraction and concentration steps directly from rare-earth-containing ores [3,4]. Looking for new sources of REEs, interest in secondary and residual deposits that contain REEs is growing. Mining residues and rock weathering, such as mining wastewaters, acid mine drainage (AMD), and regolith, are listed as potential sources of REEs, and research is under development to extract and optimize REE recovery [4,5]. In this context and considering that REEs in mining wastewaters and AMD are present in low amounts, adsorbent and ion exchange materials could be useful as part of preconcentration and/or purification processes, which makes the evaluation of those types of materials for REE capture from secondary sources interesting. This review explores fundamental aspects of REEs to understand the origin and characteristics of secondary sources and explore options for REE recovery from those sources using commercial ion exchange resins and synthetic polymeric materials to finally focus on cellulose-based materials with the aim of considering cellulosic materials as a viable and sustainable alternative to commercial resins and synthetic materials.

## 2. Chemistry of REEs

**Figure 2:**
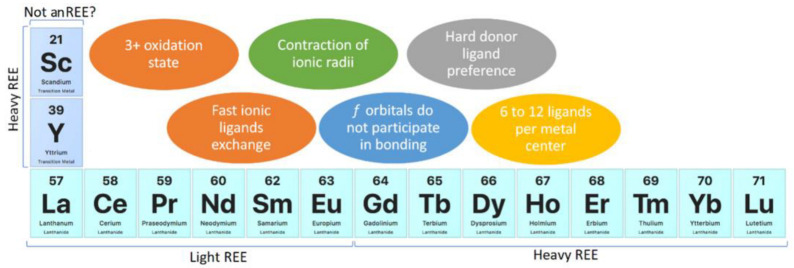
REE chemistry highlights.

The chemical properties of REEs may be divided in two categories: geochemical and chemical. Chemical properties allow the prediction of the behavior of REEs, and their understanding should be considered in the design of REE compounds and materials. In the case of REE recovery, these properties should be considered for the development of adsorbents or ion exchange materials. REEs are considered reactive elements; they are as reactive as group II elements from the periodic table and are more reactive than transition metals [2]. The most stable oxidation state of REEs is 3+, and in aqueous media, they are present as [Ln(H_2_O)_9_]^3+^ and [Ln(H_2_O)_8_]^3+^ (Ln: lanthanide), whose coordination numbers depend on the REEs’ ionic radii [2]. REE complexes show between 6 and 12 ligands per metallic center, which are influenced by steric effects rather than crystal field splitting and a preference for hard donor ligands, such as oxygen [2,4,5]. Furthermore, REEs’ nitrogen compounds, such as amino and pyridine complexes, do not have enough stability due to the basification of aqueous media, which are produced by the equilibria of nitrogenated compounds acting as weak Brønsted–Lowry bases. The preference of REEs for OH^−^, which is a harder donor ligand than nitrogen from amino compounds, causes precipitation as Ln(OH)_3_ instead of complexation with nitrogenated ligands in aqueous media [2]. REEs’ ionic compounds show fast ligand exchange in aqueous media [2]. Although REEs have *f* orbitals, those orbitals are shielded by *5s* and *5p* orbitals, and they do not participate directly in bonding [2,5]. REEs are subdivided into light REEs and heavy REEs, where light REEs refer to La, Ce, Pr, Nd, Sm, and Eu, and heavy REEs refer to Gd, Tb, Dy, Ho, Er, Tm, Yb, and Lu, together with Sc and Y [3,5]. Although this classification could be considered quite controversial because it initially seems focused on REEs’ atomic weight, Sc and Y are the lightest REE elements. Nevertheless, Sc and Y are considered heavy REEs because they share some chemical properties with heavier REEs due to lanthanide contraction of ionic radii [2,3,4,5]. The lanthanide contraction of ionic radii is caused by poor *f*-orbital screening, pushing electrons closer to the atomic nucleus and leading to similar physical and chemical properties of REEs [2,3,5]. As previously mentioned, Sc could be excluded from REEs due to its chemical properties, which differ enough from other REEs [2,3,4,5] (Figure 2).

The geochemical properties of REEs refer to the behavior of the elements in the earth’s crust, and their understanding is crucial for understanding REE distribution, mining, and mobility in the environment. The geochemical behavior of REEs follows all chemical properties already mentioned. REEs are not found as native elements, and they are always part of minerals and ores [4,5]. REE minerals and ores are primarily formed by magmatic and hydrothermal processes [4,5]. These processes allow the accumulation of REEs in carbonatites, alkaline, and peralkaline igneous rocks [4,5]. REEs are lithophile (rock-loving) elements, occurring as oxides, silicates, phosphates, and oxyhydroxide salts [3,4,5]. REEs can substitute other cations of similar radius, which change the structure of minerals [3,4,5]. Erosion, rock weathering of primary sources and other mineral deposits, together with mine exploitation, contributes to the mobility of REEs in the environment and the formation of secondary REE deposits [4,5].

## 3. Applications of REEs

Currently, portable devices of domestic usage, technological advances, and industrial needs have increased the demand for REEs, which are also used in applications related to medical sciences and renewable energy sources and, in China, as fertilizers in agriculture (Table 1). In electronics, REE compounds are part of screens and electronic displays. These compounds emit light at specific wavelengths upon application of electric fields, which are responsible for producing a wide range of colors [3,4,6]. Technological applications of REEs are related to light emission or transmission, as they are part of forming coherent light emission in lasers or total internal reflection compounds used in fiber optics [3,4,5,6]. Additionally, REE compounds could be part of high-temperature superconductors [4,6]. In medicine, nuclear resonance imaging uses Gd-based contrast agents to improve imaging resolution [4]. Radioactive REE isotopes, such as Y-90 and Sm-153, have been used as part of cancer treatment therapies [4,5,6]. REE applications related to renewable energies use Nd in permanent magnets, which are applied in low-friction turbines [3,4,5,6]. Additionally, La has been used as a component in anodes for classic Ni–metal hydride batteries [4,5,6]. Chemical processes use REE-based catalytic columns for petroleum cracking [3,4,5,6]. Other REE-based catalysts are used to produce plastics and organic compounds [3,4,5,6]. REE oxides are used as pigments in ceramics and glass. The Chinese agroindustry uses REE nitrates and oxides as fertilizers, which increase both crop quality and yields [3,4,6]. 

## 4. Sources and Recovery of REEs

### 4.1. Primary Sources of REEs

The main source of REEs comes from the mining industry. REEs are extracted mainly from the minerals bastnasite [Ln(F,OH)CO_3_], monazite [(Ln,Th)PO_4_], and xenotime [(Y,Ln)PO_4_] (Ln: lanthanide). Although 250 minerals ores contain REEs [3,4,5], only approximately 10 minerals and ores contain REEs in considerable amounts [3]. The abundance of individual REEs varies depending on the mineral source. As a typical example, it is known that monazite is rich in lighter REEs, while xenotime is rich in heavier REEs [2,3,4]. REEs with an even atomic number are more abundant (see Figure 1) [1,2,3,4]. Additionally, REE abundance diminishes with the increasing of atomic weight [1,2,3,4]. Despite their abundance, deposits of REEs of economic interest, due to deposit size or REEs’ amount present in minerals, are uncommon, and most of them are in specific geographic areas [3,4,5,6,7,8,9]. From primary sources, REE extraction processes include the acid digestion of REEs containing ores and the use of solvent extraction to selectively separate REEs and obtain the respective REE oxides [3,4,5,6,7,8,9,10,11,12]. 

### 4.2. Secondary Sources of REEs

Processes for REE recovery from secondary sources, such as mining wastewaters, AMD, and regolith, are under development in pilot scales [5,6,7,8,9,10,11,12]. Mining wastewaters are acidic and are produced due to the active exploitation of mines that contain economically relevant minerals, and in some cases, they could contain REEs [7,8]. AMD is a phenomenon that occurs when heavy metal- and sulfur-containing rocks and minerals are exposed to air and humidity. Sulfur oxidation due to the exposure of oxygen in the air produces sulfuric acid, which corrodes minerals and rocks. When REEs are present near AMD sites, the acidifying nature of AMD increases the mobility of REEs in soils and waters, which also happens with mining wastewaters [9,10]. On the other hand, REEs’ regolith formation is associated to the mobility of REEs from mining wastewaters or AMD. When REEs are liberated from acidic waters to groundwaters, the presence of clay minerals allows the sorption of REEs present, which accumulate over time, although this process is still not well understood [11,12]. REEs have been found in concentrations up to 100 times more than naturally found amounts in soils and waters surrounding REE mines and processing plants [5,6,7,8,9,10,11,12]. 

### 4.3. Recycling and Recovery of REEs

REE recycling and recovery processes could be also considered secondary sources of REEs and are related to three possible REE sources: electronic devices, industrial processes, and industrial waste. Currently, REE recovery is focused on industrial processes, where magnets, lamps, petroleum cracking catalysts, and batteries are recycled [10,13]. REE recovery from industrial waste has been proposed due to their economic significance. REE recovery processes from mining and other types of extractive industries have been proposed and implemented [10,11,12,13,14,15,16]. Pilot-scale studies have been developed for REE recovery, and circular economy processes have been proposed for REEs [10]. These processes involve solvent extraction, precipitation, inorganic sorbents, and ion exchange resins [10,11,12,13,14,15,16]. 

The main challenge for REE recovery from recycling or secondary sources is related to the low amount of REEs present in those sources, requiring the implementation of new techniques and materials related to preconcentration steps, in which selectivity toward REEs is highly desirable [5,6,7,8,9,10,11,12,13,14,15,16,17]. The recovery of REEs from water has the following two main approaches: precipitation and sorption [5,6,7,8,9,10,11,12,13,14,15,16,17]. Therefore, adsorbent and ion exchange materials have been considered a practical approach to capture and recover REEs as preconcentration steps [10,16,17] due to ease of scalability, low energetic requirements, and high efficiency [18], which could allow selective, rapid capture and recovery of REEs from secondary sources, such as mining wastewaters and AMD. The advantages of REE recovery from secondary sources are the same advantages offered by the usage of sorption or ion exchange materials already described.

## 5. Environmental Impact and Health Effects of REEs

Although the increase in RE demand has also increased REE production and related waste, knowledge about the environmental impact and health effects from exposure to REEs has not been extensively studied, which differs from other heavy metals [3,4,5,19,20]. Some concerns about REE-containing ores refer to the presence of radioactive elements, such as Th or U, which are also present in REE mineral deposits and storage areas, where high levels of nuclear radiation have been measured [3,4,5]. Concerns exist about Gd presence in water sources across Europe and the US due to its usage as a contrasting agent in medicine, which is excreted in urine, and it has been found in water treatment plants [4]. The effects of REE exposure in biological organisms have been studied, which have shown necrosis, hepatic and pulmonary damage, behavioral changes in rats and mice [19], development impairment in fish and toxicity in marine organisms [19,20,21,22], and plant growth inhibition due to disruption in nutrient uptake and plant metabolism [3,4,19]. Effects in plants are topics of concern considering the usage of REE oxides and nitrates in Chinese agriculture and should be discussed, especially considering that REE usage in agriculture adds a route of REE exposure to human beings and animals [22]. In humans, REE effects have been reported in some enzymes found in the gastrointestinal tract, which could cause malabsorption, indigestion, and protein deficit [3,19,20]. REEs also accumulate in the liver and bones [3,19,20]. REE accumulation has been reported in brain tissue, which could cause neurological and nephrological damage [19,20]. Pneumoconiosis and lung diseases have been reported in REE mining workers [3,4,5,20]. REE exposure causes cognitive impairment, bone alteration, tissue toxicity, and male sterility [3,4,17,20,22]. Increments in oxidative stress markers after REE exposure have also been reported, which could be dangerous for REE long-term exposure, although these effects have been used as tools for novel cancer treatments [20]. Critical REE toxicity values in human beings have been estimated for Sm (III) between 500 and 2000 mg/L [22].

The US EPA has a strict regulation for REE mining and processing, which has halted REE mining operations due to environmental concerns [3,4]. China has the largest REE mining production, and evidence points to the presence of REEs in water and soils near REE mining facilities and noted health effects on human populations near those facilities [3,4,5,22]. However, there is no clarity about maximum exposure limits for REEs in waters for human consumption from environmental regulators and agencies, although limits to REE exposure have been proposed based on background concentrations of REEs in pristine environments [23]. Moreover, if industrial and mining waste derived from exploitation and product manufacture related to REE is considered, the lack of regulations about REE exposure and growing evidence suggesting that REE exposure could be hazardous [3,4,19,20,21,22], it is possible to expect an environmental and/or human disaster in the midterm. Considering the facts exposed in this section, recovering REEs from secondary sources, such as mining wastewaters and AMD, could reduce the environmental and human population damage caused by REE exposure, which could be seen as a desirable advantage in the search for more sustainable economic extraction processes.

## 6. Factors Affecting REE Uptake

### 6.1. Sorption and Functional Groups

**Figure 3:**
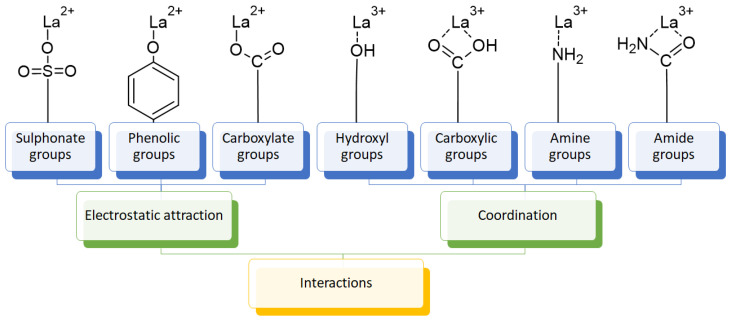
Interactions between REE cations and common functional groups included in polymeric materials for REE uptake.

The usage of polymeric materials dominates REE uptake research, and most materials studied for this purpose are polymeric in nature [24,25,26]. In general terms, REE sorption processes for batch experiments have been described using adsorption isotherms. Adsorption is defined as the increase in the concentration of a chemical substance in an interphase in which one of the phases is a solid substance. The nature of the surface interactions determines the resistance of the retained substance (adsorbate) for being removed from the solid surface (adsorbent) [27,28]. Langmuir and Freundlich isotherms are probably the most used models for this purpose. Although other adsorption isotherm models have also been evaluated for experimental results, the Langmuir and Freundlich isotherms are preferred for their simplicity. A Langmuir model states that all active sites are equivalent and have the same affinity for the adsorbate (monolayer model), while a Freundlich model states that the active sites have different affinities for the adsorbate, and they are occupied starting from the sites with the highest affinity for the adsorbate and constantly generating new active sites with lower affinity (multilayer model) [27]. Applying those models for REEs, which are found as cations in aqueous media, should consider interactions between REEs and a proper moiety present in the surface of the material. The nature of these interactions could be by means of electrostatic attraction (ionic interactions with a moiety of opposite charge) or through an electron free pair present in the moieties of the surface of the adsorbent material (coordination) [27,28,29] (Figure 3). Polymers and their derivatives incorporate into their surface SO_3_^−^, -OH, COOH, -NH_2_, -NH, and -MO functional groups and moieties [24,25,26,27,28]. Due to the chemical properties of REEs, ionic-type interactions are preferred due to REEs’ high charge (3+) [27,28,29]. Most of the materials evaluated for REE uptake fit into the Langmuir sorption model due to the fact that most polymeric materials evaluated include only one type of functional group, thus presenting active sites with the same affinity for REE cations. Incorporation of different functional groups or combining different polymers in the same material could make possible the application of the Freundlich sorption model in some cases [27,28]. 

### 6.2. Polymeric Material Structure

Most, if not all, materials used for REE uptake are porous. The porosity of a material affects the kinetics and the effective surface of the material: more pores in a material could lead to better REE uptake due to a higher sorption surface and faster REE sorption [28]. In terms of pore size, smaller pores could increase the surface of the material [17,28]. Likewise, particle size affects the effective sorption surface of the material, and smaller particles have a higher effective surface for REE uptake, although the use of nanomaterials for REE uptake should be carefully addressed due to the tendency of nanomaterials to aggregate [17,28]. Taking advantage of the described effects of the material structure should be considered in the design of new materials for REE uptake. Mesoporous materials (pore sizes between 3–10 nm) are described for REE uptake, showing high surface areas (between 350–650 m^2^/g) and acceptable REE uptake capacities [28,29]. Nevertheless, using more porous materials and smaller particle sizes does not guarantee higher REE uptake because the nature of the interactions between the surface of a material and REEs impacts the most the REE uptake capacity, as could be observed in the reviewed works next to this section. In general terms, if the same type of material with different porosity and/or a different particle size is evaluated, the statement about a more porous and smaller particle size means a more effective sorption surface and a higher REE uptake could be applied. The surface area of the material is commonly determined by the application of nitrogen at 77 K adsorption/desorption isotherms, in which the BET model is applied [17,27].

## 7. Synthetic Polymeric Materials for REE Uptake

Ion exchange resins have been widely used to capture REEs; however, the performance and selectivity of those commercial resins have limited their usage to preconcentration steps and primary separation in the whole process of REE extraction and recovery [27,28,29]. Polymeric adsorbents/ion exchange materials and composites, which include polymeric organic and inorganic hybrid materials, have been developed to achieve better performance and selectivity toward REE recovery (Table 2). These materials incorporate carbonyl and nitrogen groups, showing the maximum capture capacity of REEs at low acidic or neutral pH values.

### 7.1. Commercial Ion Exchange Resins

Commercial cation exchange resins, such as Dowex 50WX8, Lewatit MDS 200H, and Purolite C160, have low capture capacity for REEs in batch studies, although researchers have found selectivity toward light REEs [16]. Interestingly, those resins were evaluated at low pH values, up to 3.4, which is a characteristic pH value for mining wastewaters and AMD [16]. In another study, Dowex SCX HCR-S/S resin showed high capture capacity for Eu (III) coming from radioactive waste of nuclear-related processes [30]. It should be noted that evaluated resins are based on polystyrene–divinylbenzene (PS–DVB), which has a sulfonic group in their structure, and possibly due to differences in manufacturing processes, which impact resin grain size and the degree of crosslinking, they have different capture capacities for the same REE cations. Furthermore, commercial cationic exchange resins by themselves have low capture capacity for REEs, and they require modifications to improve their REE uptake. According to the information given in those selected works, it is unknown whether the presence of other metal cations could interfere with REE capture in those resins, although ion exchange theory suggests high selectivity toward REE cations due to their high charge (3+) [27,43]. Moreover, selectivity toward light REEs in those resins could be explained by the polarizability of the ions evaluated. Light REE cations are larger ions than heavy REE cations due to lanthanide contraction of ionic radii, giving light REEs greater polarizability and, thus, more affinity in commercial ion exchange resins [27,43]. 

### 7.2. Novel Synthetic Polymeric Materials

Other synthetic resins different from the commercial ones have also been studied for REE uptake from aqueous media. Using polystyrene (PS), which is one of the most used polymers in commercial ion exchange resins, together with poly (hydroxamic acid) to form an interpenetrating polymer network (IPN), the capture capacity for REEs increases up to 200 mg/g for Ce (III), 150 mg/g for La (III), and 120 mg/g for Y [31]. This drastic increase in the capture capacity, compared with other commercial ion exchange resins, could be explained by the chelating effect of hydroxamic acid, which has been used as a flotation agent in REE mining [44]. The nature of IPN, where two crosslinked polymers are entangled [45], gives the resulting material a better availability of the functional polar groups found in hydroxamic acid possibly due to repulsion with the polystyrene network. PS–DVB resin with diglycolamic acid groups shows a similar capture capacity for REEs to commercial ion exchange resins for Dy (III) at low pH values [32]. Interestingly, diglycolamic acid captures REEs only by a chelating effect and not through ion exchange because, at the low pH values evaluated, carboxylic acid moieties are protonated. Resorcinol–terephthalaldehyde resin is abundant in carbonyl groups due to the presence of terephthalaldehyde and deprotonated hydroxyl groups due to treatment with NaOH during synthesis. It shows a capture capacity of 70 mg/g for Eu (III) at pH 4.0. According to researchers, it is a nontoxic resin due to its nonhazardous starting materials [33]. However, the aldehyde moiety present in this resin could be oxidized to a carboxylic acid, and protonation of hydroxyl groups under the conditions found in mining wastewaters and AMD should be considered because it could change the REE uptake properties. It has also been reported that using a lanthanide ion imprinted polymer (IPP) of poly(4-vinylpyridine), which is also a chelating resin, captures approximately 130 mg/g of REEs [34]. It seems that the capture capacity in this type of material increases for heavier REEs, probably because of the lanthanide contraction, which makes imprinted ion cavities found in the material more available for the size of the ions evaluated. It should be noted that poly (4-vinylpyridine) was evaluated at pH 6.0, which keeps nitrogen groups present in the pyridine ring deprotonated. Based on REE recovery from mining wastewaters or AMD, nitrogen-chelating resins are not suitable for this type of application, and carboxylic-acid-based chelating resins should be preferred because they work under acidic conditions near the pH of mining wastewaters and AMD.

### 7.3. Loaded Synthetic Polymeric Materials

Synthetic organic polymers supported on silica have been studied for RE uptake. Poly-diglycolamide supported on silica particles shows similar capture capacities for REEs to commercial ion exchange resins, although it seems more selective toward REEs at low pH values [35]. Another silica-based material with polyacrylamide also shows similar capture capacities for Tb (III) to commercial resins at pH 6.0 [36]. Poly (acrylamide–sodium 4-styrenesulfonate) supported in silica composites shows up to 136 mg/g capture capacity for REEs at neutral pH, and it seems selective toward light REEs [37]. Poly (acrylamide–acrylic acid–sodium 4-styrenesulfonate) supported in silica composites shows 206 mg/g capture capacity for Gd (III) at neutral pH due to mixed functionalities of sulfonate and carboxylate groups present in the composite [38]. Interestingly, most silica-supported materials evaluated in this work show high capture capacities for REEs at pH values near neutral, which allows both chelating and ion exchange effects from carboxylic acid and nitrogenated groups present in those materials. These functional groups are also found in polymeric materials already mentioned. It seems that silica does not influence the REE capture capacity, and it only works as support media. Nevertheless, it should be pointed out that silica is an inexpensive, versatile material as a support due to its high porosity, high surface area, chemical stability, mechanical properties, and the highly advanced development of techniques associated with producing silica particles with different morphologies, sizes, and porosities, which makes silica highly attractive as support material [46,47,48,49]. 

Fe_3_O_4_ composites have been reported for REE capture as a convenient alternative to silica particles due to their magnetic properties. Poly(itaconic acid) supported in sepiolite (Fe_3_O_4_) showed up to 178 mg/g capture capacity for REEs, and they seemed selective toward heavy REEs at low acidic to neutral pH values [39]. 2-Ethylhexyl phosphonic acid mono-2-ethylhexyl (PC88A)–doped silica functionalized β-cyclodextrin supported in Fe_3_O_4_ performs better than commercial resins capturing REEs, having up to 64 mg/g capture capacity for REEs. It showed selectivity toward REEs in the presence of other metal cations, such as Na (I), Mg (II), Ca (II), Ni (II), and Zn (II), at low acidic pH values [40]. Poly (methyl methacrylate–glycidyl methacrylate–ethylenimine)-grafted-Fe_3_O_4_ microspheres produced by means of a novel irradiation-induced grafting method showed capture capacity up to 189.8 mg/g for Ce (III) at pH 6.0 [41]. Although the performance of these particles is promising, the acidic conditions found in AMD or mining wastewaters decrease the amount of Ce (III) captured to approximately 40 mg/g [41] due to the protonation of amino coordinating groups present in the material. In general terms, the usage of Fe_3_O_4_ particles in composites has some advantages: they share some properties with silica particles, such as high porosity and high surface area, and their magnetic properties allow easy recovery of the composites from aqueous media, which are especially useful features in batch applications [50]. Additionally, effects in surface morphology have been observed, which favors interaction with functional groups associated with Fe_3_O_4_-based composites. It has been reported that Fe_3_O_4_-based composites with organic functional moieties or inorganic modifiers usually have high heavy metal uptake [51,52,53,54], which is also observed with REE capture, as shown in selected works. 

Furthermore, the use of polysaccharide derivates and composites has also been reported for REE capture. Chitosan-grafted poly(acrylic acid) supported in attapulgite composites shows a high capture capacity for REEs, up to 333 mg/g for La (III) at low-acidic to neutral pH values [42]. Although these preliminary results could suggest REE selectivity toward light REEs, it could be interesting to evaluate composites with heavier REEs, such as Eu (III) and Gd (III). It should be noted that β-cyclodextrin and chitosan are polysaccharides coming from renewable sources, and likewise with synthetic organic polymers, as shown in selected works mentioned, those biopolymers could be successfully incorporated into composites. Composites that incorporate those biopolymers show similar performance to synthetic polymeric materials toward REE uptake, making them a suitable and less hazardous option to design and implement materials and techniques related to REE capture from mining wastewaters, AMD, and other sources.

## 8. Cellulosic Materials for RE Capture

**Figure 4:**
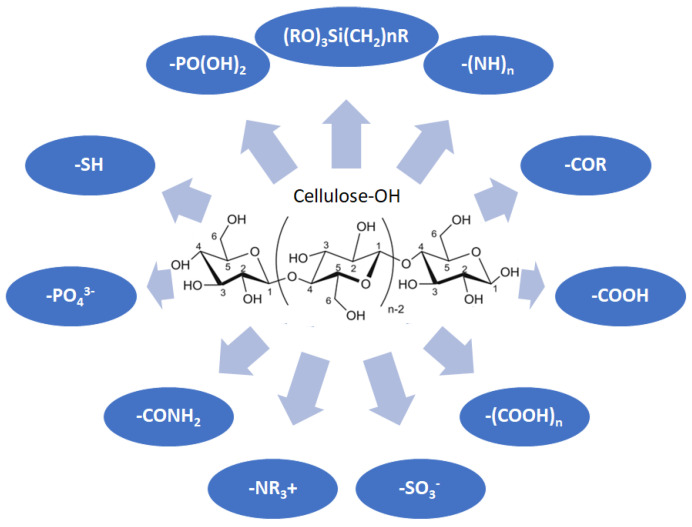
Chemical structure and modifications of interest for cellulosic-based materials.

Cellulose is a biopolymer composed of glucose units bonded by glycosidic bonds β-1,4 (Figure 4). It is the most abundant biomass-derived substance on earth, and it is found as an integral part of the cell walls in bacteria and plants [55,56,57]. 

Cellulose has been a substance of interest for researchers because of its abundance, availability, renewability, biocompatibility, biodegradability, and low cost. It also shows high versatility due to the following physical and chemical properties: mechanical stability, chemical stability, high surface area, and modifiable surface chemistry. Cellulosic materials could be chemically modified through incorporation of chemical groups in their structure by means of substitution of hydroxyl groups present in their structure [55,56,57] (see Figure 4). 

Cellulose and modified cellulose could be incorporated together with functional polymers and inorganic substances, forming composites and hydrogels. These hydrogels and composites could be used as adsorbent/ion exchange materials for metal ion removal from water [55,56,57,58]. Cellulosic materials have been evaluated to remove toxic heavy metal cations, such as Pb (II), Hg (II), Cd (II), and Cr (III); semiprecious metal cations, such as Ag (I); precious metals, such as Au (III), Pt (II), and Pd (IV); and less toxic metal cations, such as Cu (II), Zn (II), and Fe (III) [58,59,60,61,62,63,64,65,66,67,68,69,70,71,72,73,74,75,76,77,78]. Additionally, cellulosic materials have been studied for removing the toxic metal anions Cr (VI) and As (V) [77,78,79,80,81,82,83,84]. In most cases, cellulosic materials show high capture capacities and selectivity toward specific metal groups [58,59,60,61,62,63,64,65,66,67,68,69,70,71,72,73,74,75,76,77,78,79,80,81,82,83,84,85]. 

Recently, cellulosic materials have been studied for REE recovery through adsorption/ion exchange processes. As observed from cellulosic materials for metal cation capture from aqueous media, chemical modifications to cellulose and/or using organic functional polymers and inorganic hybrid cellulosic composites allow the capture of REEs (Table 3).

### 8.1. Low-Molecular-Weight-Molecule-Modified Cellulose Materials 

Cellulose by itself has low REE uptake, up to 6 mg/g [84]. However, thiourea functionalized cellulose captures up to 73 and 27 mg/g of Nd (III) and Eu (III), respectively [86]. Thiourea acts as a complexing agent for REEs, and many thiourea–metal complexes have been reported [100,101]. Researchers do not specify pH conditions in their experiments; however, thiourea has been studied alone as an extracting agent for REEs from acid leaching [102], which indicates the thiourea-cellulose materials could be applied in mining wastewaters or AMD, although more direct evidence is needed to support this claim. 

On the other hand, carboxylated cellulose captures up to 25 and 33 mg/g Tb (III) and La (III), which could also suggest selectivity toward light REEs [88,89]. Interestingly, when carboxylated cellulose is on the nanoscale, the capture capacity for La (III) increases considerably, up to 100 mg/g [90]. However, this capture capacity is reported when carboxylated nanofibrillated cellulose (NFC) is found as a suspension in aqueous media, and researchers also evaluated freeze-dried carboxylated NFC, reporting 59 mg/g of La (III) captured at neutral pH [90]. Substituting hydroxyl groups found in nanocrystalline cellulose (NCC) by a dicarboxylic acid and combining it with NCC as a support to form a composite allowed the capture of Nd (III) up to 264 mg/g at low acidic pH [91]. It should be noted that most experiments related to REEs and carboxylated cellulose were reported at neutral pH, which favors both coordinative and ion exchange effects for REE uptake. Based on the information previously discussed about polymeric materials for REE uptake related to carboxylic acid moieties, especially focused on the dicarboxylic acid–cellulosic material, which was evaluated in acidic conditions, it could be possible to apply carboxylated cellulosic materials for REE capture from mining wastewaters or AMD sources. However, more suitable studies should be performed to support this claim. Moreover, it should be highly recommended to include more REEs, because only a few were considered in the selected works. 

### 8.2. Polymer-Grafted Cellulose Materials

Moreover, a study involving carboxylated NCC/polyethyleneimine (PEI) s-IPN showed a capture capacity up to 120 mg/g of REEs and suggested selectivity toward heavy REEs at pH values near neutral [92]. The nature of the interaction between PEI and carboxylated NFC suggests low carboxylic acid availability due to entangled carboxylated NFC to the crosslinked PEI by ionic interactions between the carboxylic acid functionality present and ammonium groups of PEI. It seems that PEI amino chelating groups were favored due to near-to-neutral pH conditions, which keep nitrogen coordinating groups available for REE uptake. As previously mentioned, nitrogen-chelating materials are not suitable for this type of application due to the acidic conditions found in mining wastewaters and AMD.

On the other hand, poly (amidoxime)-grafted-cellulose capture up to 262 mg/g of La (III) at pH 6.0 and comparing the results for the other REEs evaluated suggest selectivity toward light REEs [93]. The reported capture capacity could be explained by the coordinative properties of the amidoxime moiety present in the graft polymer, which consists of both oxime and amide groups. Nevertheless, the possible capture capacity for REEs under acidic conditions found in mining wastewaters and AMD could be drastically lower, making amidoxime unsuitable for the intended application due to protonation of both amine and oxime moieties, as previously stated.

Furthermore, another study involving microcrystalline cellulose and derived grafted polymers with tetraethylenepentamine (TEPA) or poly(carboxymethyl) cellulose (PCMC) showed up to 38, 101, and 107 mg/g capture capacity for La (III), respectively, at pH 3.0–4.0 [94]. These materials are promising candidates for REE capture from mining wastewaters and AMD, especially PCMC, due to the high development related to carboxymethylcellulose, which already has many industrial and commercial applications [103]. 

### 8.3. Loaded Cellulose Materials

In a different study, graphene oxide/polyethylene glycol/carboxylated bacterial cellulose ion-imprinted polymer (IIP) composites showed up to 49 mg/g Dy (III) captured at pH 4.0 [95]. Phosphorylated NCC/carbon nanotube composites capture up to 45 mg/g of La (III) ions at pH 4.0 [96]. Carboxylated NCC/carbon nanotube composites capture up to 34 mg/g Dy (III) at pH 4.0 [97]. It seems that both graphene oxide and carbon nanotubes only work as a support for functionalized NCC and do not influence the REE capture capacity. Additionally, considering that both phosphoric and carboxylic moieties were evaluated at pH 4.0, those materials have similar performance for REE capture to carboxylated cellulose-based materials. It is highly advisable to include more REEs in future studies, as previously stated. 

Another alternative is the use of cellulose supported on silica particles, which show similar capture capacities for REEs to those reported for carboxylated cellulose. The results revealed a removal capacity up to 29 mg/g of REEs [98]. As previously discussed in REE uptake by polymeric materials, silica works as a suitable support due to its desirable properties and does not contribute to REE uptake itself. 

In addition, a different approach is observed when cellulose is supported in Al-layered double hydroxide (Al-LDH); the resulting composite shows a capture capacity for REEs up to 102 mg/g at neutral pH and seems selective toward heavy REEs [99]. However, the main drawback of these types of composites is their stability under acidic conditions found in mining wastewaters and AMD, which could cause dissolution of Al-LDH, making them unsuitable for the intended application.

## 9. Conclusions and Perspective

REEs are present in many applications (semiconductors, luminescent molecules, catalysts, batteries, and many others), and mining of REEs has increased considerably in the last decade, which is producing many environmental and human health concerns due to REE mobility in the environment from mining wastewaters and AMD.

In this review, we discussed the recovery of REEs from mining wastewaters and AMD as secondary sources using adsorbents and ion exchange materials from synthetic and natural source materials. Considerations should be taken when evaluating and proposing new materials for REE recovery from secondary sources, mainly referred to acidic pH conditions found near mining facilities and AMD, and the stability of materials under those conditions. Evaluating the selectivity of novel materials for REE capture and other possible elements that could compete with REE uptake should be taken in consideration. Furthermore, based on the evidence presented in this review, combining different functional groups in the same polymeric material or combining different polymers should be considered due to changes in selectivity toward REEs and/or improving performance of existing polymers and materials in terms of REE uptake. Carbonyl and carboxylic moieties are useful for REE capture, and their incorporation should be also considered in novel materials for REE recovery. Although the presence of amino-containing moieties could influence REE capture from aqueous media, AMD and mining wastewaters are acidic in nature. Under these conditions, nitrogen-containing groups are found protonated, which decreases the amino-containing groups’ ability to coordinate REEs, making them less suitable for REE recovery from those sources. 

According to the reported literature, cellulosic materials could have better performance capturing REEs than commercial ion exchange resins. Cellulose can be chemically modified and increase its removal capacity of REEs. In addition, the composite materials of biopolymers, such as cellulose, and synthetic polymers increase the capture capacity of REEs. Considering the evidence presented in this review, the development of this novel class of materials in recovery systems for REEs is important and could be considered, for example, in preconcentration steps.

In conclusion, recovering REEs from secondary sources using cellulose-based materials is a useful approach for the application of a circular economy in REE mining, and it could reduce the potential environmental impact and human health effects caused by REE exposure due to the liberation of these elements in localities near REE sources and mining facilities.

## Figures and Tables

**Table 1:** Applications of REEs.

Application Field	REEs Involved	Uses
Electronics	La, Eu, Gd	Screen, personal computers, mobile phones, silicon chips, LED diodes, fluorescent lamps
Technology	La, Pr, Nd, Sm, Eu, Tb, Dy, Ho, Er, Yb	Lasers, masers, fiber optics, radars, nuclear reactors, lamps, high-reflectance glass, high-temperature superconductors
Medical sciences	Y, Eu, Gd, Tm, Lu, Sm	X-ray apparatus, nuclear resonance imaging, cancer treatments
Renewable energies	La, Pr, Nd, Eu, Tb	Electric cars, turbines, batteries
Chemical and industrial processes	La, Ce, Pr, Nd, Yb, Er, Lu	Catalysts, steel manufacture, magnets, pigments, colorants, oxidizers, plastics, catalytic converters, catalytic columns
Agriculture	La, Ce, Pr, Nd	Fertilizers, crop yield and crop quality enhancers

**Table 2:** Polymeric materials for REE capture.

Polymeric Material	Functional Groups Involved	Ion Captured	Capacity q (mg/g)	Conditions Temperature (°C, pH)	Ref.
Dowex 50WX8	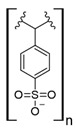	Y (III)	11.2	25, 3.4	[16]
		La (III)	25.4		
		Ce (III)	23.3		
		Nd (III)	21.1		
		Dy (III)	16.9		
		Gd (III)	17.7		
Lewatit MDS 200H	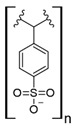	Y (III)	10.6	25, 3.4	[16]
		La (III)	26.8		
		Ce (III)	24.9		
		Nd (III)	20.9		
		Dy (III)	16.2		
		Gd (III)	17.9		
Purolite C160	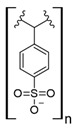	Y (III)	10.0	25, 3.4	[16]
		La (III)	28.7		
		Ce (III)	26.4		
		Nd (III)	25.5		
		Dy (III)	13.0		
		Gd (III)	13.8		
Dowex SCX HCR-S/S	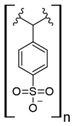	Eu (III)	161.9	25, 4.5	[30]
Interpenetrating polymer network (IPN) resin polystyrene-poly (hydroxamic acid)	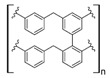 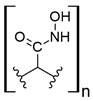	La (III)	150.0	25, 4.0	[31]
		Ce (III)	200.3		
		Y (III)	120.9		
Polystyrene/divinylbenzene/diglycolamic acid	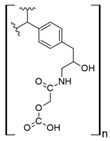	Dy (III)	17.8	25, 1.0–3.0	[32]
Resorcinol–terephthalaldehyde resin	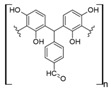	Eu (III)	70.0	25, 4.0	[33]
Poly(4-vinylpyridine)–Schiff base lanthanide ion–imprinted polymer (Ln-IIP)	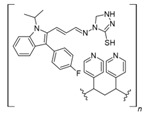	Pr (III)	125.3	25, 6.0	[34]
		Nd (III)	126.5		
		Sm (III)	127.6		
		Eu (III)	128.2		
		Gd (III)	129.1		
Silica-supported poly-diglycolamide	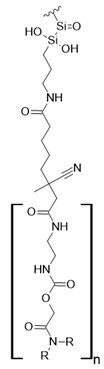	La (III)	29.0	25, 2.0–3.0	[35]
		Ce (III)	32.0		
		Pr (III)	33.0		
		Nd (III)	35,0		
		Sm (III)	39.0		
		Eu (III)	42.0		
		Gd (III)	51.0		
		Tb (III)	48.0		
		Dy (III)	51.0		
		Ho (III)	52.0		
		Er (III)	51.0		
		Tm (III)	50.0		
		Yb (III)	48.0		
		Lu (III)	49.0		
Polyacrylamide@perlite (70%–75% SiO_2_)	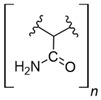	Tb (III)	24.2 29.4	25, 6.0	[36]
Poly(acrylamide–sodium 4-styrenesulfonate)@SiO_2_	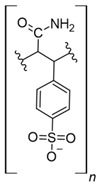	La (III),	136.0	25, 5.0	[37]
		Ce (III)	132.0		
		Nd (III)	124.0		
		Eu (III)	108.0		
Poly(acrylamide–acrylic acid–sodium 4-styrenesulfonate)@SiO_2_	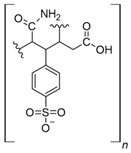	Gd (III)	206.6	25, 6.0–7.0	[38]
Poly(itaconic acid)@sepiolite Fe_3_O_4_	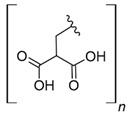	Ce (III)	106.3	35, 4.0–7.0	[39]
		Nd (III)	151.2		
		Er (III)	178.5		
2-ethylhexyl phosphonic acid mono-2-ethylhexyl (PC88A) doped silica functionalized β-cyclodextrin@Fe_3_O_4_	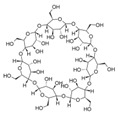	Y (III)	64.2	25, 5.0	[40]
		Nd (III)	49.2		
		Eu (III)	51.1		
		Gd (III)	40.9		
Poly(methyl methacrylate–glycidyl methacrylate–ethylenimine)-grafted-Fe_3_O_4_	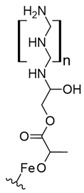	Ce (III)	189.8	45, 6.0	[41]
Chitosan-grafted-poly (acrylic acid)@attapulgite (Mg_2_Al_2_)Si_8_O_20_(OH)_2_·4H_2_O	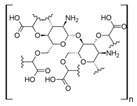	La (III)	333.3	25, 4.0–7.0	[42]
		Ce (III)	243.9		

**Table 3:** Cellulosic materials for REE capture from aqueous media.

Cellulosic Material	Functional Groups Involved	Captured Ion	Capacity q (mg/g)	Conditions T° (°C, pH)	Ref
Nutshell cellulose	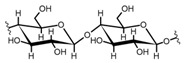	Eu (III)	5.7	25, 4.0	[86]
		Gd (III)	5.1		
		Sm (III)	6.1		
		La (III)	6.0		
Thiourea functionalized cellulose	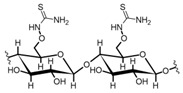	Nd (III)	73.0	25, -	[87]
		Eu (III)	27.0		
Carboxylated cellulose	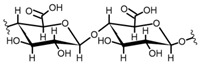	Tb (III)	25.2	25, 5.0	[88]
Carboxylated cellulose	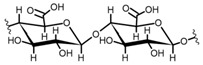	La (III)	33.7	25, 6.0	[89]
Carboxylated nanofibrillated cellulose (NFC)	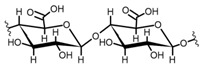	La (III)	100.7	25, 7.0	[90]
Dicarboxylated cellulose/NCC	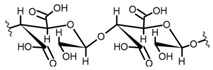	Nd (III)	264.0	25, 4.0–6.0	[91]
Carboxylated NCC/polyethyleneimine (PEI) s-IPN	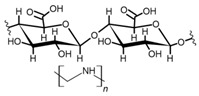	La (III)	84.8	25, 5.4–6.5	[92]
		Eu (III)	101.7		
		Er (III)	120.2		
Poly(amidoxime)-grafted-cellulose	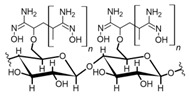	La (III)	262.0	25, 6.0	[93]
		Ce (III)	255.0		
		Pr (III)	244.0		
		Nd (III)	241.0		
Microcrystalline cellulose Tetraethylenepentamine-g-cellulose Poly(carboxymethyl)-cellulose	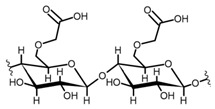	La (III)	38.0 101.0 107.0	25, 3–4	[94]
Graphene oxide/polyethylene glycol/carboxylated bacterial cellulose IIP	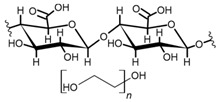	Dy (III)	49.9	25, 4.0	[95]
Phosphorylated nanocrystalline cellulose (NCC)/carbon nanotubes	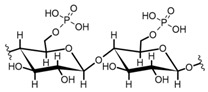	La (III)	45.9	25, 4.0	[96]
Carboxylated NCC/carbon nanotubes IIP	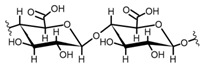	Dy (III)	34.0	25, 4.0	[97]
Cellulose@SiO_2_	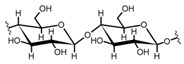	Eu (III)	24.2	25, 6.0	[98]
		La (III)	29.4		
Cellulose@Zn/Al layered double hydroxide (LDH)	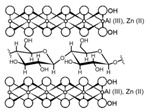	Y (III)	102.2	25, 7.0	[99]
		La (III)	92.5		
		Ce (III)	96.2		

## Data Availability

Not applicable.
